# Degradation during Mixing of Silica-Reinforced Natural Rubber Compounds

**DOI:** 10.3390/ma17020341

**Published:** 2024-01-10

**Authors:** Ammarin Kraibut, Wisut Kaewsakul, Kannika Sahakaro, Sitisaiyidah Saiwari, Jacques W. M. Noordermeer, Wilma K. Dierkes

**Affiliations:** 1 Department of Rubber Technology and Polymer Science, Faculty of Science and Technology, Prince of Songkla University, Pattani 94000, Thailand; a.kraibut@utwente.nl (A.K.); kannika.sah@psu.ac.th (K.S.); 2 Sustainable Elastomer Systems, Department of Mechanics of Solids, Surfaces and Systems, Faculty of Engineering Technology, University of Twente, P.O. Box 217, 7500 AE Enschede, The Netherlands; 3 Elastomer Technology and Engineering, Department of Mechanics of Solids, Surfaces and Systems, Faculty of Engineering Technology, University of Twente, P.O. Box 217, 7500 AE Enschede, The Netherlands; w.kaewsakul@utwente.nl; 4 Netherlands Natural Rubber Foundation (Rubber-Stichting), 3051 JG Rotterdam, The Netherlands; jacquesnoordermeer@gmail.com

**Keywords:** natural rubber, degradation, chain scission, silanization

## Abstract

The optimal mixing conditions for silica-filled NR compounds dictate the need to proceed at a high temperature, i.e., 150 °C, to achieve a sufficient degree of silanization. On the other hand, natural rubber is prone to degradation due to mechanical shear and thermal effects during mixing, particularly at long exposure times. The present work investigates NR rubber degradation during mixing in relation to prolonged silanization times. The Mooney viscosity and stress relaxation rates, bound rubber content, storage modulus (G’), and delta δ were investigated to indicate the changes in the elastic/viscous responses of NR molecules related to rubber degradation, molecular chain modifications, and premature crosslinking/interaction. In Gum NR (unfilled), an increase in the viscous response with increasing mixing times indicates a major chain scission that causes a decreased molecular weight and risen chain mobility. For silica-filled NR, an initial decrease in the Mooney viscosity with increasing silanization time is attributed to the chain scission first, but thereafter the effect of the degradation is counterbalanced by a sufficient silanization/coupling reaction which leads to leveling off of the viscous response. Finally, the higher viscous response due to degradation leads to the deterioration of the mechanical properties and rolling resistance performance of tire treads made from such silica-filled NR, particularly when the silanization time exceeds 495 s.

## 1. Introduction

Silica-reinforced tire tread rubber compounds have been growing in use since their successful introduction to improve tire performance in order to achieve fuel-efficient and safe-driving tires [1]. However, the improvement in the compatibility with and good dispersion of silica in a rubber matrix is challenging in compound processing. This is due to the different polarity causing incompatibility between silica and nonpolar rubber, i.e., natural rubber (NR), styrene-butadiene rubber (SBR), and butadiene rubber (BR), which are mostly used in tire treads. To enhance the compatibility and good dispersion of silica in these rubber matrices, bifunctional organosilanes are essential ingredients in silica-filled compounds. In addition, a mixing temperature of about 135–155 °C [2,3,4,5] is necessarily applied to ensure a complete reaction of the silanol groups of the silane and the hydroxyl groups on the silica surface, so-called “silanization”. The main governing factors of tire tread performance are determined by the intrinsic properties of the rubbery components, the elastomers, of the tire compounds. This is an important consideration when choosing a material for utilization in a tire tread application. Natural rubber is preferentially employed in truck and aircraft tire tread applications due to its excellent mechanical properties, high elasticity, and low heat build-up. Numerous studies of silica-reinforced NR-based tire tread compounds have been published. Kaewsakul et al. [4] reported the optimum mixing conditions for silica-filled NR to achieve desirable mechanical properties and the rolling resistance of tire tread compounds. The appropriate mixing process was necessarily operated at high temperatures in the range of 135–150 °C and a silica–silane–rubber mixing interval of 10 min. The properties of vulcanizates were found to correlate with the processing temperature during the mixing of such silica-filled NR compounds. It was concluded that to achieve optimal final properties, the mixing of silica-filled NR compounds needed to be operated at the earlier mentioned high temperatures in order to reach sufficient silanization and proper silica–silane–rubber coupling [3,4,5,6,7]. However, the mixing process at such high temperatures is prone to the thermal–oxidative degradation of NR [8,9].

The degradation of NR is a free radical process that can be initiated by several modes, i.e., oxidative, thermal, and chemical. During mixing, the degradation of NR can occur due to mechanical effects: high shear forces during mixing generate heat and initiate rubber chain breakage. Consequently, unstable chain radicals are formed. These radicals are the initiators for further degradation and lead to chain scission, resulting in a decrease in the molecular weight and so deteriorated mechanical properties. All associated factors, chemicals, oxygen, temperature, and time, can contribute to the degradation of NR [10]. Kumar et al. [8] investigated the change in the molecular properties of NR films through thermo-oxidation. The extension of the exposure time of NR films at 100 °C led to a decrease in the molecular weight as a result of chain scission. A study on unfilled NR degradation by the evaluation of physical property changes reported that reduced tensile and tear strength were also due to a decreased NR molecular weight [11]. Additionally, rubber degradation via decreased rubber molecular weight was observed with increasing mastication time and reaction time during the mechanical, chemical, and photooxidative degradation of epoxidized natural rubber (ENR) [12]. The degradation of unfilled NR compounded at a high mixing temperature of 180 °C was studied. It was reported that an increase in the mixing time from 5 to 10 min at a certain mixing temperature led to the significant degradation of such unfilled NR compounds [9].

In our previous work, degradation phenomena during mixing were reported for silica-filled NR compounds with the main governing factor being temperature [6]. The degradation during mixing was analyzed by changes in the dynamic mechanical response. At high discharge/dump temperatures above 150 °C, the Mooney stress relaxation rate and dynamic mechanical properties measured in a frequency sweep indicated a significant molecular chain modification that caused a heterogeneous network and the deterioration of the mechanical properties of the vulcanizates [6]. In addition, other mixing conditions, i.e., shearing force and mixing time, may also contribute to rubber degradation. Especially, a long mixing time at the typical elevated temperature of silanization may lead to more radicals generated, thus influencing rubber degradation and the mechanical properties of rubber vulcanizates. Therefore, the present work aims to investigate the rubber degradation of silica-filled NR with different silanization times by using Gum (unfilled) NR compounds as references. Various techniques are applied to monitor rubber degradation, including structural changes, viscoelastic responses, and the creation of long-chain branching by using the technique of delta-delta (Δδ) as the different phase angle at a low (i.e., 0.01 Hz) and high frequency (i.e., 16 Hz). In addition, tire-related properties, i.e., the mechanical and dynamic characteristics of the silica-filled NR vulcanizates, are investigated.

## 2. Materials and Methods

### 2.1. Materials

Standard Malaysian Rubber 10 (SMR10), the NR grade mostly used in tire treads, was supplied by WEBER & SCHAER GmbH & Co. KG, Hamburg, Germany. Silica ULTRASIL 7005 with CTAB (Cetyl-Trimethyl-Ammonium Bromide) and BET (Brunauer–Emmett–Teller) specific surface areas of 171 and 190 m^2^/g, respectively, and the silane coupling agent bis-(3-TriEthoxySilylPropyl)-Disulfide (TESPD) were both obtained from Evonik, Essen, Germany. Treated Distillate Aromatic Extract oil (TDAE oil) (Vivatec 500) was obtained from Hansen & Rosenthal, Hamburg, Germany. DiPhenyl Guanidine (DPG), N-Cyclohexyl-2-Benzothiazyl Sulfenamide (CBS), 2,2,4-TriMethyl-1,2-dihydroQuinoline (TMQ), and N-(1,3-dimethylbutyl)-N′-Phenyl-p-PhenyleneDiamine (6PPD) were supplied by Lanxess Rhein Chemie GmbH, Cologne, Germany. Zinc oxide (ZnO), stearic acid, and sulfur were obtained from Merck KGaA, Darmstadt, Germany. All materials were used as received.

### 2.2. Compound Formulations

Silica-filled NR compounds were prepared by using chemicals and curatives according to typical truck tire tread formulations as given in Table 1. An unfilled NR compound or so-called Gum NR was prepared as a reference.

### 2.3. Mixing

#### 2.3.1. Silica-Filled NR Compounds

**Figure 1:**
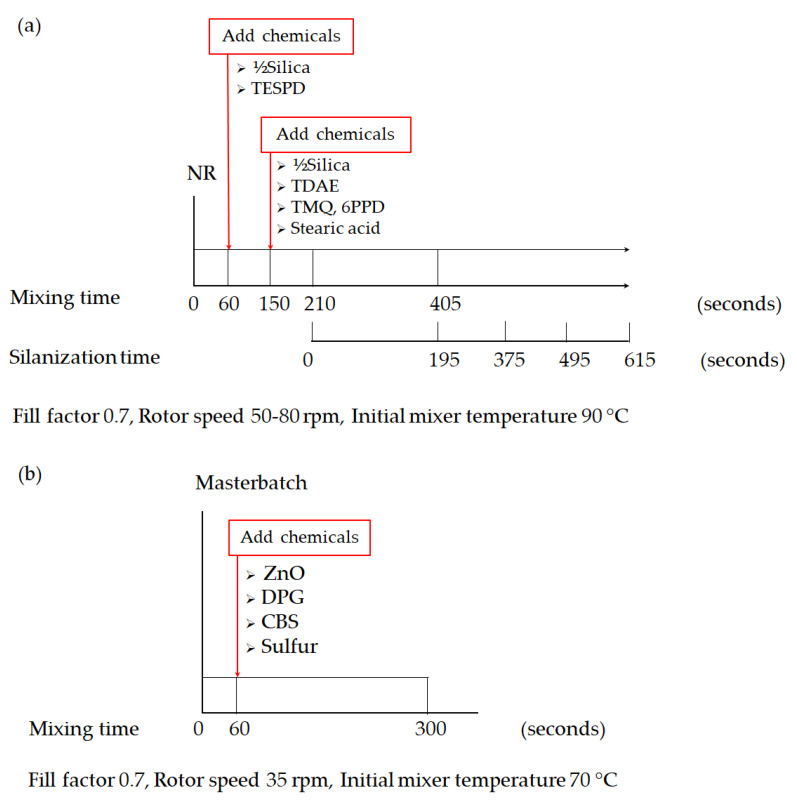
Two stages of the mixing procedure for preparing the compounds: (**a**) nonproductive step and (**b**) productive step.

The rubber and chemicals were mixed in a lab-scale internal mixer with a mixing chamber of 50 cm^3^ (Brabender GmbH & Co. KG, Duisburg, Germany) by using a fill factor of 0.7. The initial mixer temperature was set at 90 °C with varying rotor speeds from 50 to 80 rpm to achieve dump temperatures of 145–150 °C. The mixing was conducted in 2 steps according to the procedures shown in Figure 1. In the first step, the nonproductive step, NR was masticated for 60 s, and then the first half of the silica and all of the silane were loaded. After 150 s of mixing, another half of the silica together with TDAE, stearic acid, TMQ, and 6PPD were loaded. The mixing continued for 210 s when the mixing temperature reached 140 °C and the silanization started. To ensure the completion of silanization, the mixing was continued for another 195 s to reach a total mixing time of 405 s. To examine the effect of longer mixing times at the typical elevated temperature of silanization in this work, the typical silanization time was extended to 375, 495, and 615 s. As shown in Figure 1a, after reaching the total mixing time, the compound was then sheeted out on a two-roll mill to cool down in the final procedure of the nonproductive step. Subsequently, the productive mixing step was carried out at an initial mixer temperature of 70 °C to avoid premature vulcanization or scorching during the process in the same internal mixer. After the mastication of the masterbatch for 60 s, the curatives ZnO, DPG, CBS, and sulfur were added to the mixer. This productive mixing step was completed after 300 s as shown in Figure 1b at an approximate dump temperature of 85 ± 5 °C. Finally, the compounds were sheeted out on a two-roll mill.

#### 2.3.2. Gum Reference Compounds

To be compared with the silica-filled NR, an unfilled or Gum NR was prepared and used as a reference in this study. The Gum NR compounds were also prepared in 2 steps of mixing following the mixing procedures applied for silica-filled NR as shown in Figure 1a, using the selected mixing conditions. Nevertheless, without the filler involved in the 1st mixing step, the dump temperature of the Gum compounds is lower than that of silica-filled NR. Therefore, the mixer temperature setting was adjusted to reach similar dump temperatures as found for the silica-filled NR compounds. The total mixing times of the Gum compounds were extended to be equal to the total mixing times after prolonged silanization times in the silica-filled compounds. Therefore, the term “prolonged mixing times” is used in the Gum compounds as well.

### 2.4. Characterization

#### 2.4.1. Mooney Viscosity and Mooney Stress Relaxation

The nonproductive compounds were cooled down and left overnight at room temperature. The Mooney viscosity and Mooney stress relaxation were analyzed by using a Mooney viscometer (MV200VS, Alpha Technologies, Hudson, OH, USA) according to ASTM D1646 [14]. The Mooney viscosity was tested at 4 min after preheating for 1 min and is reported as ML 1+4 (100 °C) (large rotor) for Gum and MS 1+4 (100 °C) (small rotor) for the filled NR. Note that due to the more common large rotor, some silica-filled compounds gave an initial value above the limit of the Mooney viscometer. Thus, the small rotor was used for all the filled compounds for consistency reasons. In the Mooney stress relaxation test, when the rotor of the Mooney viscometer stops after the completion of the viscosity test, the torque is detected for another 1 min. The stress relaxation of the compounds is described through a power-law model, as shown in Equation (1):

(1) $$M=k{\left(t\right)}^{a}$$

where

*M* = Mooney units (torque) after the completion of the viscosity test,
*t* = relaxation time (s),
*k* = a constant equal to the torque in Mooney units at 1 s after the disk is stopped,
*a* = exponent that determines the rate of stress relaxation.

Then, Equation (1) can be expressed in a log–log correlation, as given in Equation (2):

(2) log⁡M=a(log⁡t)+log⁡k

According to a log–log plot of Mooney units against time, the rate of stress relaxation is defined by the slope (*a*). The Mooney stress relaxation is a combination of elastic and viscous responses. A higher value of the relaxation rate indicates an increase in the viscous/elastic ratio. Numerous studies of raw rubber and rubber compounds reported that the rate of stress relaxation correlates with the structural characteristics of the rubber, such as the molecular weight, molecular weight distribution, chain branching, and gel content [6,15].

#### 2.4.2. Payne Effect

The storage shear moduli (G’) of the uncured silica-filled NR compounds obtained from the nonproductive mixing step were evaluated by using an RPA elite dynamic spectrometer (TA Instruments, New Castle, DE, USA) at a temperature of 100 °C, frequency of 0.5 Hz, and various strains in the range of 0.56–100%. The Payne effect, which indicates filler–filler interactions, was calculated by the differences in storage shear moduli at a low strain (0.5%) and high strain (100%) [16].

#### 2.4.3. Bound Rubber Content (%)

In total, 0.20 g by weight of the uncured nonproductive compounds were immersed in 20 mL of toluene for 3 days at room temperature in an air atmosphere. Thereafter, the sample was removed from the toluene, dried at 80 °C in a vacuum oven for 24 h, and weighed. The *bound rubber content* (BRC) was calculated according to Equation (3) [17]:

(3) $$Bound\;rubber\;content\;\left(\%\right)=\frac{W_{ext}-W_{f}}{W_{p}}\times 100$$

where

*W_ext_* = the weight of the sample after extraction,
*W_f_* = the weight of silica in the original sample,
*W_p_* = the weight of the polymer in the original sample.

#### 2.4.4. Dynamic Properties as a Function of Frequency

The dynamic properties of the uncured nonproductive compounds were analyzed by using the RPA elite dynamic spectrometer as well. A frequency sweep mode was used at various frequencies in the range of 0.1 to 16 Hz with a strain amplitude of 5% and at a temperature of 100 °C. At this strain magnitude, the linear viscoelastic regime of the material was observed. The change in the phase angle (δ) with frequency was employed to characterize long-chain branch formation as described by Booij [18].

#### 2.4.5. Dynamic Properties as a Function of Temperature

Dynamic mechanical properties of the rubber vulcanizates were characterized in tension mode by using a DMA Eplexor 2500 (NETZSCH-Gerätebau GmbH, Ahlden, Germany) with various temperatures in the range of 20 °C to 80 °C at a constant frequency of 10 Hz and a 0.1% strain. The rubber vulcanizate sample dimensions were 6 × 4 × 2 mm^3^. The tangent of the phase angle (tanδ) at 60 °C was taken as indicative of the rolling resistance of the tire treads [19].

#### 2.4.6. Tensile Properties

The Gum NR and silica-filled NR compounds were vulcanized into 2 mm thick sheets by using a Wickert Laboratory Press (WLP1600, Wickert Maschinenbau GmbH, Landau in der Pfalz, Germany) at 150 °C and 100 bar by using their optimum cure times (t_c,95_). The vulcanized sheets were die-cut to dumbbell-shaped specimens (type 2). Tensile tests were carried out with a Zwick tensile tester (model Z1.0/TH1S) at a crosshead speed of 500 mm/min according to ASTM D412 [20]. The tensile modulus at a 300% strain as well as stress and elongation at break were analyzed.

## 3. Results and Discussion

### 3.1. Mooney Viscosity and Mooney Stress Relaxation

**Figure 2:**
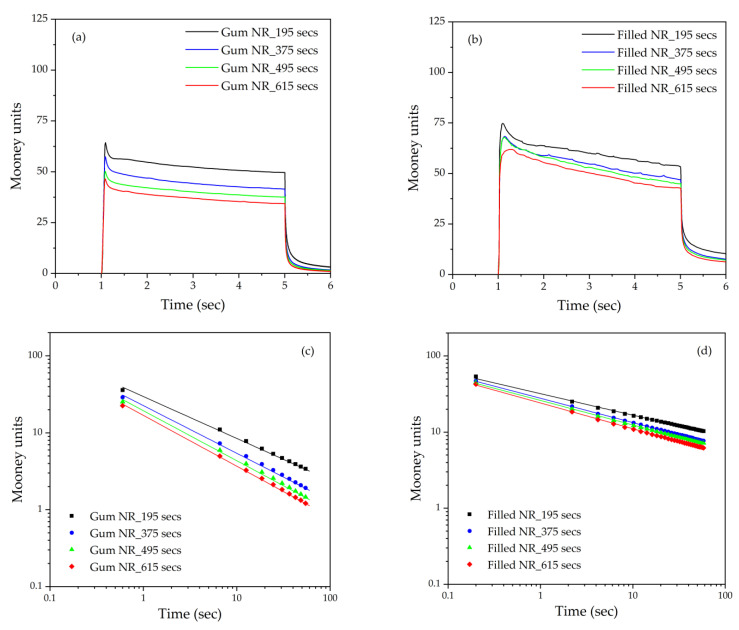
Mooney curves (**a**,**b**) and double logarithmic plots of Mooney stress relaxation (**c**,**d**) of Gum (ML 1+4, (100 °C)) and filled (MS 1+4, (100 °C)) NR compounds subjected to various mixing or silanization times.

**Figure 3:**
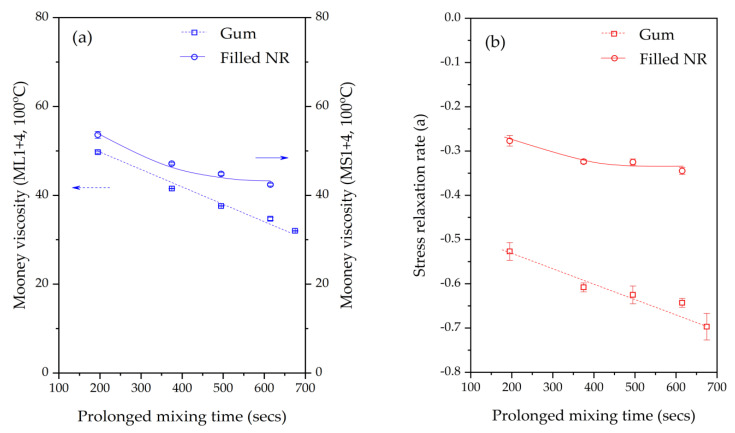
Mooney viscosity (**a**) and Mooney stress relaxation rate (**b**) of the Gum (ML 1+4, 100 °C) and filled NR (MS 1+4, 100 °C) compounds subjected to various mixing or silanization times. **Note** the negative representation of the Mooney stress relaxation rate (**a**): the lower the value, the higher the stress relaxation.

The Mooney curves of the Gum and silica-filled NR compounds with various mixing/silanization times are shown in Figure 2a,b. In both the Gum and silica-filled NR compounds, the Mooney viscosities decrease with increasing mixing or silanization times. However, a slight difference in the decreasing trend of the Mooney viscosity is observed between the Gum and silica-filled compounds, as can be seen in Figure 3a. In Gum NR, an increasing mixing time results in a decrease in the Mooney viscosity in a linear trend. This marks an increase in the rubber molecular mobility, which may be attributed to chain scission during the extended mixing times. In addition to shorter chains or the lower molecular weight generated, chemical changes on NR chain terminals are expected after the oxidative degradation. The occurrence of the carbonyl groups on the NR molecules subjected to prolonged mastication was clearly demonstrated by means of FTIR [8]. In Gum NR, the prolonged mixing time decreased the Mooney viscosity and increased the viscous response uniformly, which indicates that the changes were mainly governed by physical processes. In the case of the filled NR, a different trend is seen: the Mooney viscosity first decreases with an increasing silanization time for 375 s like for Gum. With further increases in the silanization time to 495 and 615 s, the decreasing trend in the Mooney viscosity becomes leveled off. The initial decrease in the Mooney viscosity with the increasing silanization time to 375 s can be attributed to a combination of rubber molecular chain scission and decreased filler–filler interactions due to the enhanced hydrophobation of the silica. Longer silanization times result in the completion of the silanization and coupling reactions, therefore showing only a slight decrease in the Mooney viscosity. The carbonyl chain ends of the degraded chains should also help to enhance the rubber–silica interaction. These phenomena correspond well with the Mooney stress relaxation rates shown in Figure 2c,d: the double logarithmic plots of the Mooney stress relaxation for the Gum and filled NR compounds subjected to the various mixing or silanization times. Similar differences in changes in the Mooney stress relaxation rates with increasing mixing or silanization times are seen. The somewhat steeper slope for Gum NR is again attributed to a higher viscous vs. elastic response due to molecular chain scission, whereas the lower slope in the filled NR indicates a higher elastic response due to the rubber–filler interaction. In Gum NR, the increase in the Mooney stress relaxation rates with increasing mixing times results in a more or less linear trend line like with the Mooney viscosity itself, as shown in Figure 3b (note that the negative representation of the numbers is for the ease of comparison with the Mooney viscosity data). In the silica-filled NR, the Mooney stress relaxation rates increase first and then level off with an extended silanization time over 375 s. Further increasing the silanization to 495 and 615 s results in leveled-off Mooney stress relaxation rates, which can be attributed to the degradation being counterbalanced by the silanization/hydrophobation of the silica/silane filler system.

### 3.2. Payne Effect of Silica-Filled NR

**Figure 4:**
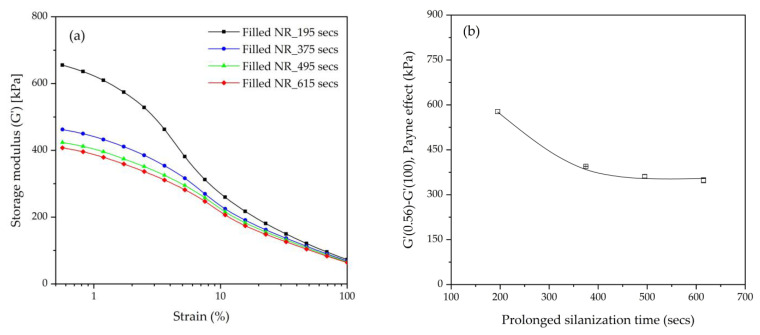
Storage modulus against the percentage of strain (**a**) and Payne effect (**b**) of filled NR compounds subjected to various silanization times.

The storage moduli (G’) as a function of strain for the silica-filled NR compounds with various silanization times are shown in Figure 4a. At low strain, a strong decrease in the storage modulus with an increasing silanization time from 195 s to 375 s is clearly observed. Further increases in the silanization time to 495 and 615 s result in a lower decrease in the storage modulus. Generally, the decreasing storage modulus at a low strain indicates the breakage of the filler network, commonly reported to correspond with filler–filler interactions: the Payne effect [16]. Therefore, the clear reduction in the low-strain storage modulus with an increasing silanization time from 195 s to 375 s indicates a decrease in filler–filler interactions. This is attributed to a progressing degree of silanization/hydrophobation with an increasing silanization time from 195 s to 375 s. Applying a longer silanization time, i.e., 495 and 615 s, only gives a slight further decrease in the storage modulus. The decreasing trend of the storage modulus is reduced as the silanization comes to completion, and possibly some short chains are still generated by polymer chain scission.

The difference in the storage modulus between low and high strains, i.e., 0.56–100%, is commonly employed to quantify the Payne effect as an indicator for the degree of the filler–filler interaction. The Payne effects are given in Figure 4b. A drastic decline in the Payne effect with an increasing silanization time up to 375 s is observed, indicating a strong decrease in the filler–filler interaction due to the hydrophobation of the silica surface. It is a prerequisite for proper silica dispersion in the rubber matrix. This is in accordance with several previous reports [3,4,5]. Extended silanization times over 375 s lead only to a slight further decrease in the Payne effect, which marks again saturation in the silanization and optimum coupling reaction between the silane and silica [4].

### 3.3. Bound Rubber Content and Storage Modulus at 100% Strain

**Figure 5:**
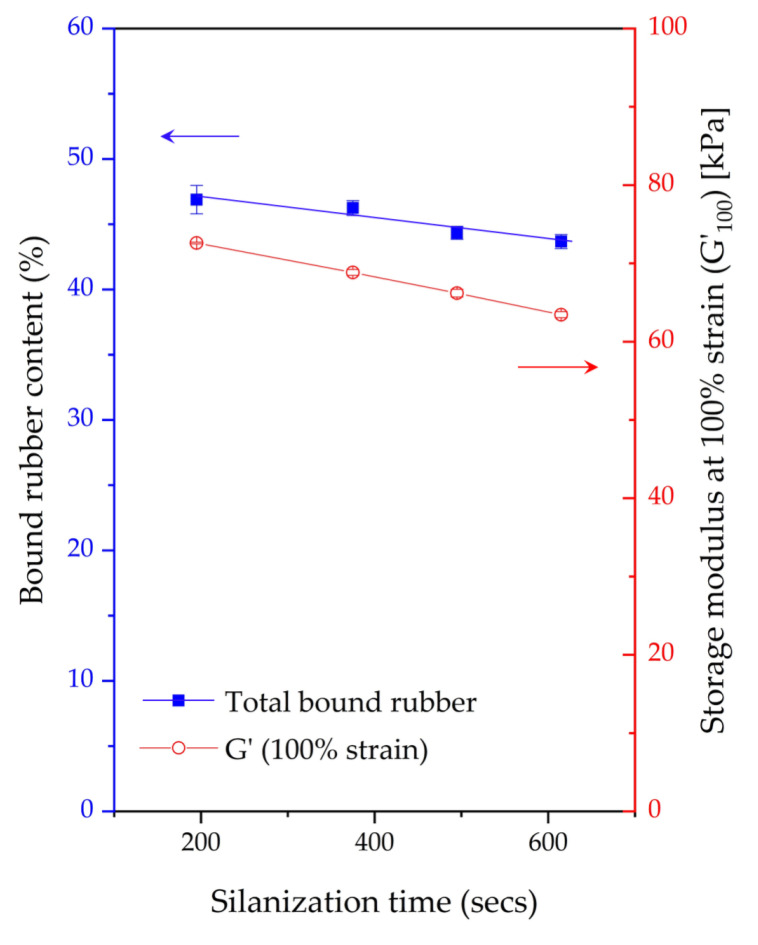
Bound rubber content and storage modulus at 100% strain of filled NR compounds subjected to various silanization times.

**Figure 6:**
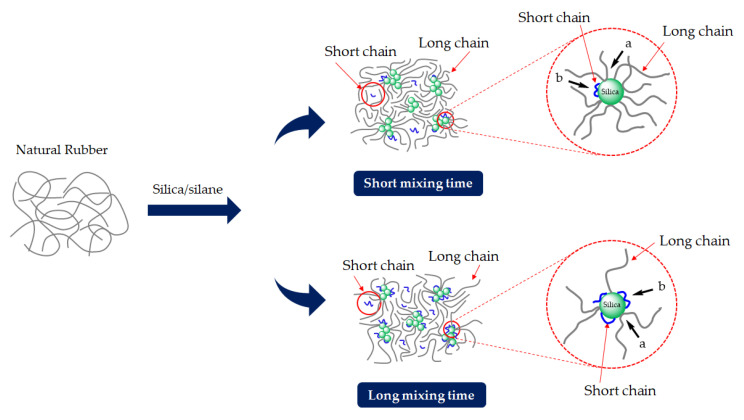
Proposed concept of bound rubber chains directly contacting the filler surface; single-contact rubber chain (a) and multiple-contact rubber chain (b) [17,21].

Generally, in silica-filled rubber compounds, bound rubber represents molecular chains that are bonded to the silica surface by both physical and chemical interactions and are not able to be extracted from the compounds by a good solvent, i.e., toluene for NR [17,21]. Bound rubber content basically measures the filler–rubber interaction. Figure 5 shows the total bound rubber content after different silanization times. The bound rubber content apparently decreases with increasing silanization time. This is again clearly related to degradation during mixing, wherein the rubber molecular chains break down. This leads to a higher number of short molecular chains or lower average molecular weight [4]. In addition, the higher number of short chains occurring at longer silanization times causes a lower reactivity of the rubber toward the silica surface and also less rubber chain entanglements. This corresponds well with the previous study, which stated that a change in the rubber molecular weight from chain breakage led to a reduction in the bound rubber content. Subsequently, the increase in short chains causes multiple-segment adsorption onto the filler surface, leading to decreased effectiveness in forming bound rubber [17]. This is attributed to the short molecular chain fragments having a higher number of reactive moieties interacting with active silane on the silica surface. Figure 6 is a schematic depiction of the possible bound rubber interactions on the filler surface consisting of single-contact rubber chains and multiple-contact rubber chains. When a segment of the rubber chain attaches to the filler surface, a whole molecule becomes part of the bound rubber. In the case of multiple-segment adsorption, two or more active sites on the filler are occupied by the same molecule without increasing the bound rubber. This includes the formation of bonds between neighboring aggregates (interaggregate multiple-segment adsorption). An increase in chain scission with a prolonged silanization time causes the multiple-segment adsorption of rubber molecules on the silica surface, resulting in the decreased effectiveness of bound rubber formation.

The storage modulus at 100% strain is an indicator of the amount of crosslinks in the rubber matrix after the filler–filler network is completely broken down at a high strain [22]. These crosslinks consist of chemical rubber–filler interactions, sulfur crosslinks of rubber chains due to the added sulfur in the compound, and rubber chain entanglements. However, since nonproductive compounds were used in this test, the crosslinks indeed consist primarily of rubber–filler interactions and rubber chain entanglements. According to the analytical data on the polysulfane chain length distribution of silane coupling agents reported by Luginsland [23], the typical chemical structure of TESPD consists of a majority of the sulfur chain length with two atoms (S_2_), accounting for 85–88 wt%, followed by three atoms of sulfur (S_3_) at 11 wt%. As a result, TESPD primarily forms mono- and disulfide bonds (96–99%) in the silica–TESPD–rubber coupling. Only a small portion of 1–4 wt% appears to be polysulfane chain lengths ranging from S_4_ to S_7_. Therefore, the sulfur donor from TESPD can be considered negligible in its effect. In Figure 5, the storage modulus at 100% strain decreases with increasing silanization times, leading to a higher degree of chain scission again, resulting in a decrease in the NR molecular weight, an increase in the number of short chains, and a decrease in rubber chain entanglements. Therefore, all these contribute to the lower G’ at this high strain. This phenomenon is clearly associated well with decreasing bound rubber content as discussed before.

### 3.4. Dynamic Properties

A tool to further investigate the formation of long-chain branches due to the degradation of rubber is the delta-delta (Δδ) method developed by Booij [18]. This method was originally based on the principle of the molecular relaxation of viscoelastic materials at low- vs. high-oscillating frequencies. At low frequencies, e.g., 0.016 Hz, the phase angle (δ) is highly sensitive toward branched structures: tending to 90° for prevailing viscous behavior at no long-chain branching to low values for prevailing elastic behavior due to long-chain branching. At higher frequencies, e.g., 16 Hz, the phase angle is primarily determined by short-range segmental motions in the polymers, irrespective of whether they are branched or not. This fundamental concept is applied to the difference in the phase angle values calculated from Δδ: δ(0.01)–δ(16) in terms of frequency (Hz) as a measure for the width of the molar mass distribution or the degree of long-chain branching [18]. A decrease in the Δδ value indicates more branching formation and lower chain mobility.

**Figure 7:**
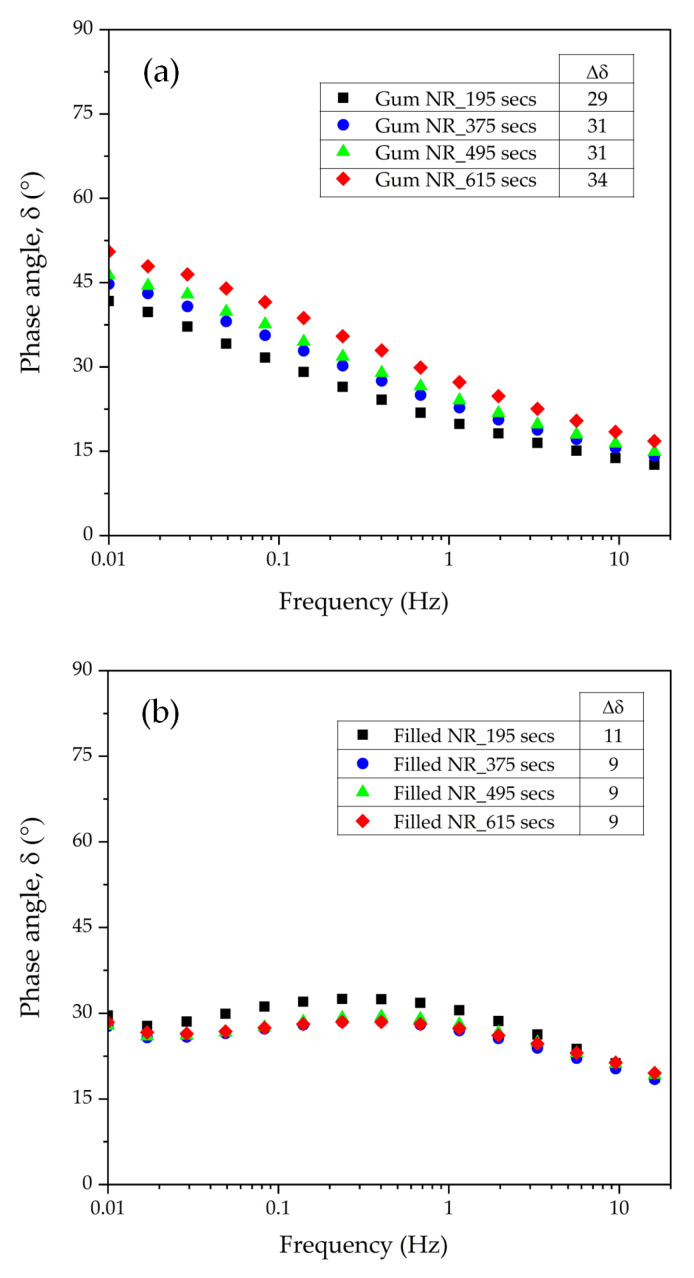
Phase angles as a function of the frequency and delta-delta (Δδ) values for uncured compounds of Gum NR (**a**) and filled NR (**b**) differing in mixing or silanization times.

**Figure 8:**
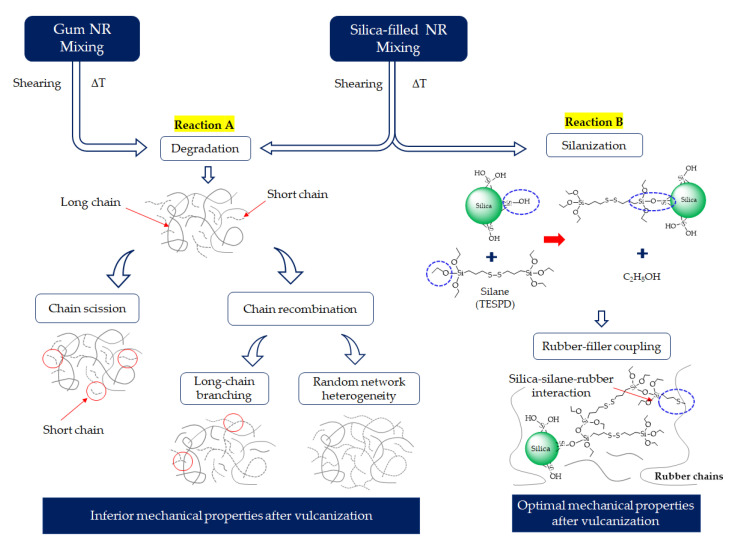
Proposed two reactions conjointly occurring during mixing of Gum and silica-filled NR compounds.

The phase angles as a function of the frequency and Δδ values for the Gum and silica-filled NR compounds at various mixing or silanization times are shown in Figure 7. In the case of the Gum reference compounds, an increase in Δδ is observed with an increasing mixing time. It marks increased chain mobility, which implies an increase in the degree of chain scission with longer mixing times. However, a different trend of the Δδ is observed for the silica-filled NR compounds. The Δδ first decreases with an increasing silanization time from 195 to 375 s. The leveling off of the Δδ values is observed with prolonged silanization over 375 s. A simplified reaction scheme proposed for the degradation mechanism of Gum and silica-filled NR during mixing is given in Figure 8. During the mixing of the Gum NR compounds, mechanical shear and heat lead to the breakdown of the rubber chains and generate unstable carbon-chain radicals causing degradation. The short molecular chain fragments with reactive moieties can result in two reactions: chain scission and chain recombination through the formation of active chain segments. Based on the results for Gum NR, the increased chain mobility with mixing time marks a higher rate of chain scission than recombination or long-chain branch formation. However, in the case of silica-filled NR, two other reactions, silanization and coupling reactions, take place, as shown in Figure 8. The coupling reactions increase the rubber–filler interaction, leading to stronger elastic behavior and decreasing chain mobility. Despite the use of TESPD with a mostly sulfur chain length of two atoms and the sulfur donor being considered negligible as described earlier, a coupling reaction can still take place during high-temperature mixing under a prolonged silanization time. Based on the results, increasing the silanization time from 195 to 375 s leads to a decrease in Δδ. This reduction in Δδ is related to the increase in rubber–filler interactions by coupling (Reaction 8B) causing a restriction of the movement of the molecular chains. Accordingly, the leveling off of the Δδ values is observed for silanization over 375 s. This implies the sufficient silanization and coupling reactions of the silica-filled NR compounds at silanization times over 375 s.

### 3.5. Mechanical Properties

**Figure 9:**
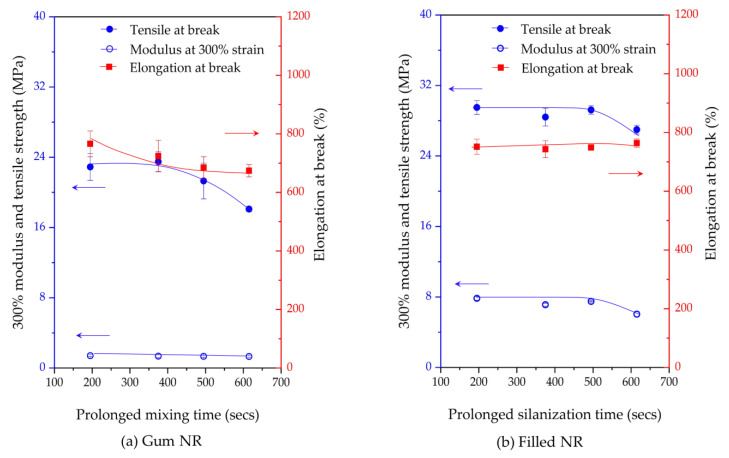
Modulus at 300% strain, tensile strength, and elongation at break for Gum NR (**a**) and filled NR (**b**) vulcanizates subjected to various mixing or silanization times.

Figure 9 shows the modulus at 300% strain, tensile strength, and elongation at the break of the Gum and silica-filled NR vulcanizates prepared with different mixing or silanization times. For the Gum vulcanizates, the modulus at 300% strain remains largely unchanged with an increasing mixing time, while a decreasing tensile strength and elongation at break are observed. This can be attributed to chain scission occurring at longer mixing times. Therefore, short molecular chains deteriorate the Gum vulcanizates’ mechanical properties. A remarkably different trend is observed for the silica-filled NR vulcanizates: an unchanged elongation at break is observed with increasing silanization times (Figure 9b). Also, the tensile strength and modulus at 300% strain stay unchanged when the silanization time is raised from 195 to 495 s but thereafter reduces sharply for silanization times over 495 s, as shown in Figure 9b. In the Gum vulcanizates, only the chemical network generated by sulfur vulcanization is involved, and chain scission clearly deteriorates the mechanical properties, while in the silica-filled NR vulcanizates, both the sulfur crosslinking network and silica–silane–rubber coupling network are present and therefore display different behaviors compared to Gum.

In particular, NR suffers from long mixing (i.e., more than 495 s) by degradation under such a high temperature required for the silanization reaction, as evidenced by the changes in the mechanical properties of the Gum NR vulcanizates shown in Figure 9a. In silica-filled NR, the change in the mechanical properties in relation to the prolonged silanization time is less, attributed to the competitive reactions between degradation and silanization. The chain scission is compensated by a sufficient silanization and coupling reaction. Therefore, the negative effects of the chain scission on the mechanical properties by degradation are counterbalanced. However, for too long silanization times, i.e., 615 s, other reactions such as long-chain branch formation and preliminary crosslinking can occur. The visible reduction in G’ and total bound rubber content at silanization times over 615 s indicate the increase in the number of short chains via chain scission, while the silanization and coupling reactions are finalized. The formation of long-chain branching and preliminary crosslinking is created by the short chains. Such chain modifications as shown in Figure 8 via reaction A gradually promote network heterogeneity, resulting in a decreasing trend of both the modulus and tensile strength.

### 3.6. Dynamic Mechanical Properties as Indications of Tire Performance

**Figure 10:**
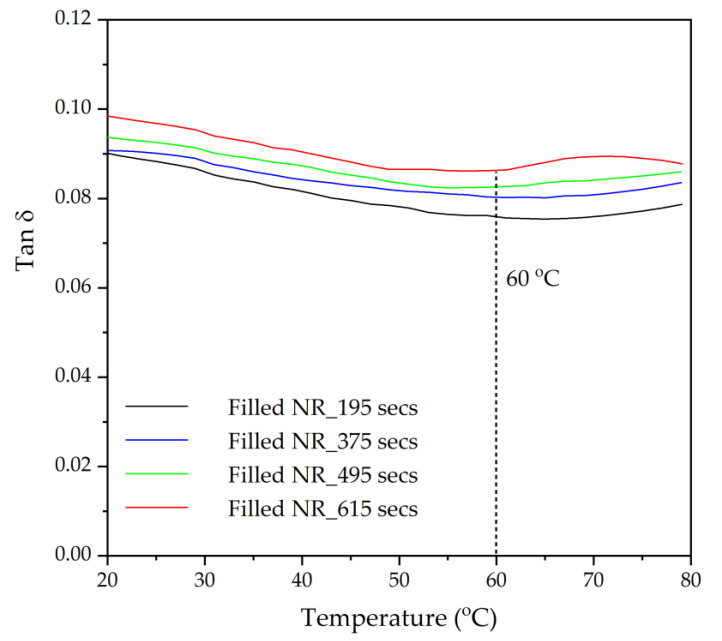
Tan δ as a function of temperature, in particular at 60 °C, as an indication of rolling resistance for filled NR vulcanizates differing in mixing or silanization time.

The loss tangent (Tan δ) as a function of the temperature of the silica-filled NR compounds with various silanization times is given in Figure 10. Tan δ represents the ratio of the viscous to elastic response for a viscoelastic material, illustrating the material’s damping ability, or in other words, the energy dissipation potential of the material. Generally, the assessment of the dynamic properties vs. temperature is applied to predict the tire performance; for instance, Tan δ at 60 °C as an indication of the rolling resistance of tire treads [19]. A lower value of Tan δ at 60 °C indicates a lower rolling resistance resulting in less fuel consumption [2,24]. Figure 10 demonstrates that the loss tangent at a temperature of 60 °C increases with increasing silanization times. This is again attributed to a rising viscous response due to the increase in short molecular chains with prolonged silanization times. An increase in the viscous response results in higher damping or energy dissipation in the rubber, causing a higher rolling resistance.

## 4. Conclusions

The influence of prolonged silanization on the degradation of Gum and silica-filled NR due to molecular chain modification was analyzed by the changes in dynamic responses. This study elucidates the proper silanization time needed for the mixing of silica-filled NR to minimize rubber degradation. The results illustrate an appropriate silanization time in the range of 375–495 s to provide sufficient silanization as well as a permissible degree of rubber degradation. For Gum NR, long durations of mixing during nonproductive compounding cause changes in the properties of the vulcanized rubber due to chain scission. The degradation or chain scission results in low molecular weight fractions as evidenced by a reduced Mooney viscosity and an increased Mooney stress relaxation rate for long mixing times. This is accompanied by a rise in Δδ, which indicates higher chain mobility. A different trend is observed for silica-filled NR, whose properties reveal a balance in degradation and silanization/coupling reactions on viscous/elastic responses at extended silanization times over 375 s. Drastic changes in the Mooney viscosity, Mooney stress relaxation, Payne effect, and Δδ demonstrate a combination of degradation and silanization at the beginning of the silanization period. But thereafter, balanced reactions between degradation and premature crosslinking or branch formation due to coupling reactions are noticed as indicated by the leveling off of those properties. The bound rubber content and storage modulus at 100% strain still showed an increase in short chains with continually prolonged silanization times. Therefore, the mechanical properties of the filled NR vulcanizates slightly decrease with an increasing silanization time up to 495 s but are more pronounced with a further increase over 495 s. The strongly reduced tensile strength and modulus at 300% strain are therefore most likely due to abundant short chains and the resulting network heterogeneity. Moreover, the higher degree of chain scission with a longer duration of silanization negatively influences the dynamic properties at 60 °C, indicative of the rolling resistance of a tire tread made from silica-reinforced NR.

## Figures and Tables

**Table 1:** Rubber formulations.

Ingredients	Amount (phr, Parts per Hundred Rubber)	
	Gum NR	Filled NR
Natural rubber (SMR10)	100.0	100.0
Silica (ULTRASIL 7005)	-	55.0
Silane (TESPD) ^a^	-	5.0
Process oil (TDAE)	-	8.0
Zinc oxide	3.0	3.0
Stearic acid	1.0	1.0
TMQ	1.0	1.0
6PPD	2.0	2.0
DPG ^a^	1.1	1.1
CBS	1.5	1.5
Sulfur	1.5	1.5

^a^ Amounts of TESPD and DPG were calculated according to the following equations [13]: TESPD (phr) = 0.00053 × Q × A; DPG (phr) = 0.00012 × Q × A, where Q is the amount of silica (phr) and A is the CTAB specific surface area of the silica (171 m^2^/g).

## Data Availability

Data are contained within the article.
